# Supplementary material for: Distributed Bayesian networks reconstruction on the whole genome scale
Source: PeerJ. 2018 Oct 19;6:e5692. doi: 10.7717/peerj.5692 (PMC6197044; doi:10.7717/peerj.5692)
Supplement: Supplemental Information 1 [file peerj-06-5692-s001.zip › bnfinder-2.2/doc/doc_all.pdf]

BNfinder documentation

Release 2.0

Norbert Dojer, Paweł Bednarz, Agnieszka Podsiadło i Bartek Wilczyński

March 26, 2018

Contents

1 Users manual **3**
1.1 Installation . . . 3
System requirements . . . 3
1.2 Usage . . . 3
bnf . . . 3
bnf-cv . . . 5
bnc . . . 6
1.3 Input format for structure learning . . . 7
Preamble . . . 7
Experiment specification . . . 8
Experiment data . . . 9
1.4 Input format for classification task . . . 9
Experiment specification . . . 9
Experiment data . . . 9
1.5 Output formats . . . 9
Suboptimal parents sets . . . 9
SIF format . . . 10
BIF format . . . 11
Python dictionary . . . 11
Standard output . . . 12
Classification results . . . 12

2 Tutorial **12**
2.1 Synthetic examples . . . 12
Simple static network . . . 12
Simple dynamic network . . . 14
Setting priors . . . 15
2.2 Examples of published datasets . . . 15
Static Protein signalling network . . . 16
Dynamic Bayesian network . . . 17
Dynamic network containing self-regulatory loops . . . 17
Using multiple processors for faster computations . . . 18
2.3 Example of classification with BNfinder . . . 18

3 Supplementary methods **20**
3.1 Polynomial-time exact algorithm . . . 21
3.2 Minimal Description Length . . . 23
3.3 Bayesian-Dirichlet equivalence . . . 24

3.4 Mutual information test 25
3.5 Continuous variables 26
3.6 Network density control 26

1 Users manual

1.1 Installation

The BNFinder software uses setuptools, which is the standard library for packaging python software. The package itselff is available through the Python Package Index (http://pypi.python.org/pypi/BNfinder/) while more frequent releases as well as the source code are available from the project website in launchpad (http://launchpad.net/bnfinder). After downloading the archive containing the current version of BNF, you should extract it to a directory of choice. In unix-like systems you can do it by typing

```
tar -xzf bnfinder-xxx-xxx-.tgz
```

If you want to install the latest development version from launchpad, you can do it directly with bzr version management tool:

```
bzr branch lp:bnfinder
```

Once you have the sources extracted, the installation is performed by a single command

```
python setup.py install
```

in the source directory (**it may require the administrator privileges**).

This installs the BNfinder library to an apropriate location for your python interpreter, and the bnf, the bnf-cv and the bnc scripts which may be accessed from a command line.

In case you don't have administrator privileges, you can either use bnfinder without installation (by invoking commands within the downloaded directory) or by installing within user home directory:

```
python setup.py install --user
```

System requirements

BNFinder is written in pure python, so the only true requirement is the python2 interpreter (any reasonably recent version like 2.6.x or newer should do). Please note that BNFinder is not currently compatible with python3. If you are interested in porting BNFinder to python3 please let us know.

There are several packages one might need to install to have BNFinder working properly, most notably the packages scipy and fpconst are required for proper functioning of BNFinder

If you would like to generate ROC plots for classification, you will also need a working installation of the R language, the Rpy2 python package and ROCR package withing R.

1.2 Usage

bnf

The bnf script is the main part of BNfinder command line tools. Is is used for learning the bayesian network from data and can be executed by typing

```
bnf <options>
```

The following options are available:

-h, --help
print out a brief summary of options and exit

-e, --expr <file>
load learning data from `<file>` (this option is obligatory)

-s, --score <name>
learn with `<name>` scoring criterion; possible names are `BDE` (default) and `MDL`

-l, --limit <number>
limit the search space to networks with at most `<number>` parents for each vertex

-i, --suboptimal <number>
return at most `<number>` best scored parents sets for each vertex (default 1; negative values result in no limit)

-m, --min-empty <value>
for each vertex return only parents sets with relative probabilities greater than `<value>` (default 0)

-o, --min-optimal <value>
for each vertex return only parents sets with the ratio of their posterior probability to the optimal set's one greater than `<value>` (default 0)

-r, --fpr <value>
adjust g components of the scoring function to yield the expected proportion of false positive edges (type I error rate) equal to `<value>`; the procedure is switched off by default

-x, --max-permutations <number>
do not perform more than `<number>` permutations in the predetermination of type I error rate

-d, --data-factor <number>
multiply (each item of) the dataset `<number>` times (default 1); this option may be used to change the proportion between d and g components of the scoring function (see the definition of the *splitting* assumption); it has no effect when the option `-r` is also used

-v, --verbose
print out communicates on standard output

-p, --prior-pseudocount <number>
set the pseudocounts of data items with specified values of a vertex and its parents set to $\frac{\texttt{<number>}}{|\mathcal{V}|^{|\mathbf{Pa}|+1}}$ (resulting in the total pseudocount equal to `<number>`) – this method follows Heckerman et al [7]; when the option is unspecified, all pseudocounts are set to 1, following Cooper and Herskovitz [1]; pseudocounts are used as hyperparameters of the Dirichlet priors of the BDE scoring criterion and also in the estimation of the conditional probability distributions (CPDs) of learned network

-n, --net <file>
write the learned network graph to `<file>` in the SIF format

-t, --txt <file>
write the learned suboptimal parents sets to `<file>`

-b, --bif <file>
write the learned optimal Bayesian network to `<file>` in the BIF format

-c, --cpd <file>
write the learned optimal Bayesian network to `<file>` as a Python dictionary

-f, --fraction <value>
set the minimal weight of a parent to be considered in a parent set

-a, --chi <value>
set the alpha value for the chi-square distribution used in MIT score (default=.9999)

-g, --sloops
allow self-loops in Dynamic Bayesian Networks (by default self-loops are disabled)

-k, --cpu <number>
use number of processes for multiprocessing - by default 0 is used (no multiprocessing)

-j, --subset <file> | "concat"
input file with subset of variables (divided by space character); the feature is useful when the inference complexity is too high, allowing distributed usage of BNFinder; after all the variables are processed (including regulators), put resulting `genes_*` files into one folder and use `concat` instead of file name; if only particular variables are subject for parents set inference, it is advisable to simply edit input data file instead of using this argument

-q, --algorithm <name>
parallelization algorithm: `setwise` (default, uses each provided core to compute parents set of one variable before proceeding further) or `hybrid` (evenly distributes cores between variables, it is recommended to use when underlying network is supposed to be homogeneous with respect to the number of parents per variable, in cases when computational complexity is low or small parents set limit is used)

bnf-cv

The bnf-cv script is a utility to perform cross-validation tests. The script can be executed by typing:

```
bnf-cv <options>
```

The following options are available:

-h, --help
print out a brief summary of options and exit

-e, --expr <file>
load learning data from `<file>` (this option is obligatory)

-k, --cross-val-folds <number>
set the number of cross-validation folds to `k`; defaults to 10; if equal to 1, do not perform cross-validation at all

-s, --score <name>
learn with `<name>` scoring criterion; possible names are `BDE` (default) and `MDL`

-l, --limit <number>
limit the search space to networks with at most `<number>` parents for each vertex

-i, --suboptimal <number>
return at most `<number>` best scored parents sets for each vertex (default 1; negative values result in no limit)

-m, --min-empty <value>
for each vertex return only parents sets with relative probabilities greater than `<value>` (default 0)

-o, --min-optimal <value>
for each vertex return only parents sets with the ratio of their posterior probability to the optimal set's one greater than <value> (default 0)

-r, --roc <file>
write ROC curve to the given file; it will contain a curve for each cross-validation fold and one additional curve averaging remaining ones

-d, --data-factor <number>
multiply (each item of) the dataset <number> times (default 1); this option may be used to change the proportion between d and g components of the scoring function (see the definition of the *splitting* assumption);

-v, --verbose
print out communicates on standard output

-p, --prior-pseudocount <number>
set the pseudocounts of data items with specified values of a vertex and its parents set to $\frac{\texttt{<number>}}{|\mathcal{V}|^{|\mathbf{Pa}|+1}}$ (resulting in the total pseudocount equal to <number>) – this method follows Heckerman et al [7]; when the option is unspecified, all pseudocounts are set to 1, following Cooper and Herskovitz [1]; pseudocounts are used as hyperparameters of the Dirichlet priors of the BDE scoring criterion and also in the estimation of the conditional probability distributions (CPDs) of learned network

-n, --net <file>
write the learned network graph in the SIF format to <file>0, <file>1, ..., <file>(k-1) for each cross-validation fold respectively; k is the number of crossvalidation folds

-t, --txt <file>
write the learned suboptimal parents sets to to <file>0, <file>1, ..., <file>(k-1) for each cross-validation fold respectively; k is the number of crossvalidation folds

-b, --bif <file>
write the learned optimal Bayesian network in the BIF format to <file>0, <file>1, ..., <file>(k-1) for each cross-validation fold respectively; k is the number of crossvalidation folds

-c, --cpd <file>
write the learned optimal Bayesian network as a Python dictionary to <file>0, <file>1, ..., <file>(k-1) for each cross-validation fold respectively; k is the number of crossvalidation folds

-f, --fraction <value>
set the minimal weight of a parent to be considered in a parent set

-a, --chi <value>
set the alpha value for the chi-square distribution used in MIT score (default=.9999)

-x, --xaxis <name>
specify x axis of the roc curve (tpr is default); see Rocr manual for posiible options

-y, --yaxis
specify x axis of the roc curve (fpr is default); see Rocr manual for posiible options

-A, --diag
set to 0 to remove diagonal line from roc plots

bnc

The bnc script allows you to perform a classification task, using a network encoded in a file in cpd format. The script can be executed by typing:

```
bnc <options>
```

The following options are available:

-h, --help
print out a brief summary of options and exit

-c, --cpd <file>
load `<file>` with the conditional probability distribution gotten from BNfinder (this option is obligatory)

-d, --data <file>
load `<file>` with values of all regulator variables (this option is obligatory)

-m, --ml
output only the most likely value; switched off for default

-p, --prob <number>
output the probability of given class `number` (for all nodes)

-o, --out <file>
write the result of the classification to `file` (by default writes to `stdout`)

When neither `-m` nor `-p` option is given, writes out for every regulatee and experiment the python dictionary with probabilities for all classes.

1.3 Input format for structure learning

The learning data must be passed to BNfinder in a text file splitted into 3 parts: preamble, experiment specification and experiment data. The preamble allows user to specify some features of data and/or network, while the next two parts contain the learning data, essentially formatted as a table with space- or tab-separated values.

Preamble

The preamble allows specifying experiment peturbations, structural constraints, vertex value types, vertex CPD types and edge weights. Each line in the preamble has the following form:

```
#<command> <arguments>
```

Experiments with perturbed values of some vertices carry no information regarding their regulatory mechanism. Thus including these experiments data in learning parents of their perturbed vertices biases the result (see [5] for a detailed treatment). The following command handles perturbations:

#perturbed <experiment/serie> <vertex list>
omit data from experiment (serie of experiments in the case of dynamic networks) `<experiment/serie>` when learning parents of vertices from `<vertex list>`

One possible way of specifying structural constraints with BNfinder is to list potential parents of particular vertices. An easier method is available for constraints of the cascade form, where the vertex set is splitted into a sequence of groups and each parent of a vertex must belong to one of previous groups (a simple but extremely useful example is a cascade with 2 groups: regulators and regulatees). There are 2 commands specifying structural constraints:

#parents <vertex> <vertex list>
: restrict the set of potential parents of `<vertex>` to `<vertex list>`.

#regulators <vertex list>
: restrict the set of potential parents of all vertices except specified with `#parents` command or with previous or present `#regulators` command to vertices included in `<vertex list>` of previous or present `#regulators` command.

Note that structural constraints forcing network's acyclicity are necessery for learning a static Bayesian network with BNfinder.

Vertex value types may be specified with the following commands:

#discrete <vertex> <value list>
: let `<value list>` be possible values of `<vertex>`

#continuous <vertex list>
: let float numbers be possible values of all vertices in `<vertex list>`

#default <value list>
: let `<value list>` be possible values of all vertices except specified with `#discrete` or `#continuous` command (when `<value list>` is `FLOAT`, float numbers are possible values)

Values in `<value list>` may be integers or words (strings without whitespaces). When some vertices are left unspecified, BNfinder tries to recognize their possible value sets. However it may miss, in particular when some float numbers are written in integer format or when some possible values are not represented in the dataset (note that the size of the set of possible values affects the score).

The space of possible CPDs of some vertices given their parents may be restricted to *noisy-and* or *noisy-or* distributions. In this case, the sets of possible values of these vertices and their potential parents must be either $\{0, 1\}$ or float numbers. Moreover, BNfinder should be executed with the MDL scoring criterion. The following commands specify vertices with *noisy* CPDs:

#and <vertex list>
: restrict the space of possible CPDs of vertices from `<vertex list>` to *noisy-and* distributions

#or <vertex list>
: restrict the space of possible CPDs of vertices from `<vertex list>` to *noisy-or* distributions

The following commands set prior weights on network edges:

#prioredge <vertex> <weight> <vertex list>
: set the prior weights of all edges originating from vertices from `<vertex list>` and aiming at `<vertex>` to `<weight>`

#priorvert <weight> <vertex list>
: set the prior weights of all edges originating from vertices from `<vertex list>` (except specified in `<prioredge>` command) to `<weight>`

Weights must be positive float numbers. Edges with greater weights are penalized harder. The default weight is 1.

Experiment specification

The experiment specification has the following form:

```
<name> <experiment list>
```

where `<name>` is a word starting with a symbol other then `#`. It is used in some output formats as the name of the learned network (until `<name>` = `conditions`, in such case the input file name is used). The form of experiment names depends on the data type and, consequently, on the type of learned network:

- When the dataset consists of results of independent experiments and a static Bayesian network is to be learned, experiment names are words without the symbol '`:`'.
- When the dataset consists of results of time series experiments and a dynamic Bayesian network is to be learned, experiment names have the form `<serie>:<condition>`. Each serie must be ordered according to the condition times and cannot be interrupted by experiments from other series.

Experiment data

Each line of the experiment data part has the following form:

```
<vertex> <value list>
```

where `<vertex>` is a word and values are listed in the order corresponding to `<experiment list>`.

1.4 Input format for classification task

Format for classification task's input consists of two parts:

Experiment specification

This part follows exactly the same rules as when specifying the input for learning the bayesian network structure.

Experiment data

Each line of the experiment data part has the following form:

```
<vertex> <value list>
```

where `<vertex>` is a word and values are listed in the order corresponding to `<experiment list>`. In this part we specify only values for regulators. The same names for vertices as while learning the network structure should be given here.

1.5 Output formats

Suboptimal parents sets

Suboptimal parents sets are written to a file in a simple text format splitted into sections representing the sets of the parents of each vertex. Each section contains a leading line with the vertex name followed by lines representing its consecutive suboptimal parents sets. Each of these lines has the form:

```
<relative probability> <vertex list>
```

were `<relative probability>` is the ratio of the set's posterior probability to the posterior probability of the

empty parents set and `<vertex list>` contains the elements of the set. Lines are ordered decreasingly according to `<relative probability>`.

To show it by example, the section:

```
C
 2.333333  B
 1.000000
 0.592593  B A
```

reports 3 most probable parents sets of the vertex C: $\{B\}, \emptyset, \{B, A\}$. Moreover, it states that $\{B\}$ is 2.333333 times more probable than the empty set and the corresponding ratio for $\{B, A\}$ equals 0.592593.

SIF format

The SIF (Simple Interaction File), usually contained in files with `.sif` extension is the simplest of the supported network formats and carries only information on its topology. In this format, each line represents the fact of a single interaction. In our case such interaction represents the fact that one variable depends on some other variable. Each line contains three values:

- Parent variable identifier,
- Interaction label; its type depends on the argument of the `-i` option (specifying the number of reported suboptimal parents sets)
  - if =1 (default), `+/-` is reported when positive or negative correlation between variables is found
  - otherwise, the edge label represents the posterior probability of the interaction represented by the edge (under the assumption that the true parents set is among the suboptimal ones)
- Child variable identifier.

To show it by example, the file:

```
A + B
B - C
```

describes a network of the following shape:

$$A \xrightarrow{+} B \xrightarrow{-} C.$$

Running BNFinder with the same input file and $\neq$1 argument of `-i` option could result in the file of the form:

```
B 0.2254 A
C 0.1560 A
A 0.8563 B
B 0.0358 B
A 0.0247 C
B 0.9463 C
```

showing that the posterior probabilities of the interactions $A \to B$ and $B \to C$ are significantly higher than the other ones.

The main advantage of this format is that it can be read by the Cytoscape (`http://cytoscape.org`) software allowing for quick visualization. It is also trivial to use such data in one's own software.

BIF format

Bayesian Interchange Format (BIF) is a simple text format dedicated to Bayesian networks. It is supported in some BN applications (e.g. JavaBayes, Bayes Networks Editor) and may be easily converted with available tools to other popular formats (including XML formats and BNT format of K. Murphy's Bayes Net Toolbox). BNfinder writes learned networks in BIF version 0.15.

Python dictionary

A network saved in `<file>` as a dictionary may be loaded to your Python environment by

```
eval(open(<file>).read())
```

The dictionary consists of items corresponding to all network's vertices. Each item has the following form:

```
<vertex name> : <vertex dictionary>
```

Vertex dictionaries have the following items:

'vals' : <value list>

'pars' : <parent list>

'type' : <CPD type>
(only for vertices with *noisy* CPDs, possible values of `<CPD type>` are `'and'` and `'or'`)

'cpds' : <CPD dictionary>

The form of the vertex CPD dictionary depends on the vertex type. In the case of noisy CPD, the dictionary items have the following form:

<vertex name> : <probability>
which means (in the case of noisy-and/-or distribution) that the considered vertex is assigned value 1/0 with `<probability>` given all its parents but `<vertex name>` equal 1/0

In the case of general CPD, the dictionary has items of the following form:

<value vector> : <distribution dictionary>
where `<value vector>` is a tuple of parents' values and the distribution of the considered vertex given `<parent list> = <value vector>` is defined in `<distribution dictionary>` in the following way:

<value> : <probability>
means that the vertex is assigned `<value>` with `<probability>`

None : <probability>
means that the vertex is assigned with `<probability>` each of its possible values unspecified in a separate item

None : <probability>
means that given `<parent list>` equal to a value vector unspecified in a separate item the vertex is assigned each of its possible values with `<probability>`

Standard output

When BNfinder is executed from a command line with the option `-v`, it prints out communicates related to its current action: loading data, learning regulators of consecutive vertices and writing output files. Moreover, after finishing computations for a vertex its predicted best parents sets and their scores are reported and after finishing computations for all vertices BNfinder reports the score and structure of the optimal network.

Classification results

The result of `bnc` command is written to a file with the following format. First line contains only names of experiments:

```
classes <name list>
```

Next lines contain information depending on the options chosen. When `bnc` is executed with `-m` option, lines with most likely values for regulatees are printed to a file. All of them have the following form:

```
<vertex name> <ML value list>
```

When `bnc` is executed with `-p` option, for every regulatee one line with probability of being in the specified class is written to the output:

```
<vertex name> <probability list>
```

2 Tutorial

This short tutorial presents the most common possible uses of the BNfinder software. The first part of this tutorial is devoted to presenting possible options of the software and the input files on simplistic, synthetic examples. In the second part, we provide more realistic examples taken from published studies of data for inferring dynamic and static networks.

In this tutorial, we will assume that you are using the standalone BNfinder application as downloaded from http://bioputer.mimuw.edu.pl/software/bnf, however if you want, you can also try out these examples with our web-server at http://bioputer.mimuw.edu.pl/BIAS/BNFinder.

If you have any questions regarding this document or the described software, please contact us: bartek@mimuw.edu.pl or dojer@mimuw.edu.pl

2.1 Synthetic examples

This section shows on several simple networks, how to prepare datasets and set the parameters for network reconstruction with BNfinder. The examples include a simple static network, dynamic network and a network requiring setting prior probabilities.

Simple static network

The first example shows how to use BNfinder to learn a simple static Bayesian network. Let us imagine that we are analysisng cells under two conditions $P1$ and $P2$ and that we are interested whether any of the four genes: $G1, G2, G3, G4$ are responding to these conditions. We assume that the true network is depicted in Fig 1, i.e.

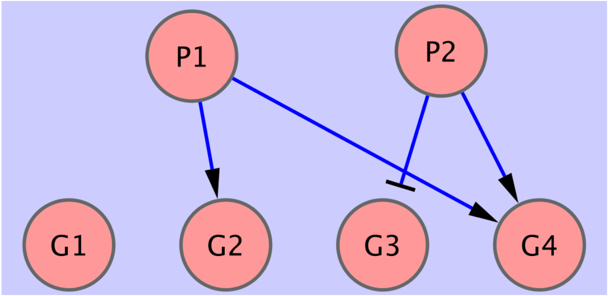

Figure 1: Very simple network consisting of 2 regulators and 4 observable regulatees.

- $G1$ is not dependent on $P1$ or $P2$,
- $G2$ is more likely to be expressed under condition $P1$,
- $G3$ is less likely to be expressed under condition $P2$,
- $G4$ is more likely to be expressed under any of the conditions $P1$ or $P2$.

We have collected 100 datapoints from this network, each consisting of both the state of conditions and the discrete state of expression of the genes. You can download the input file here data/input1.txt.

If you open the file in a text editor, please note that the first line contains the information on the assumed structure:

```
#regulators P1 P2
```

This represents the fact, that we assume that genes ($G1..G4$) can depend on conditions ($P1, P2$) and not the other way around.

You can try to run BNfinder on this file:

```
bnf -e input1.txt -n output1.sif -v
```

and you will see, that the network topology is reconstructed properly. Also the orientation of the regulatory interactions is inferred properly as you can see in the output file data/output1.sif.

You can also try to see whether the optimal network is representative for a larger set of possible suboptimal networks:

```
bnf -e input1.txt -n output1w.sif -v -i 4 -t output1.txt
```

This time, in the output file (data/output1w.sif), the edge labels represent the relative weights of different edges. In the file data/output1.txt, we can find the originally computed weights (i.e. relative probabilities – see manual for details) for all considered suboptimal sets of parents for all genes.

Instead of fixing the number of returned parents sets (option `-i 4`) you can specify thresholds for their weights and/or weight ratios to optimal weights. For example, if you wish to get for each vertex v all parents sets with weights $> \max(0.1, 0.01 \cdot w_{opt}(v))$, where $w_{opt}(v)$ denotes the weight of the optimal parents set of v, you can type:

```
bnf -e input1.txt -n output1a.sif -v -i -1 -m 0.1 -o 0.01 -t output1a.txt
```

We can also try to analyze the data for this network without discretization: data/input2.txt. In this case we need another directive to indicate that some of the dataseries are continuous:

```
#continuous G1 G2 G3 G4
```

Again if we run BNfinder on this data,

```
bnf -e input2.txt -n output2.sif -v
```

we can verify, that the output file contains correct information data/output2.sif.

Simple dynamic network

BNfinder can be used also to infer dynamic Bayesian networks from time series data. In this case it is not necessary to specify the regulators sets, because DBNs, unlike static networks do not need to be acyclic.

In the first dataset: data/input3.txt, we have 1 serie of 20 consecutive measurements of gene expression from gene network depicted in Fig. 2.

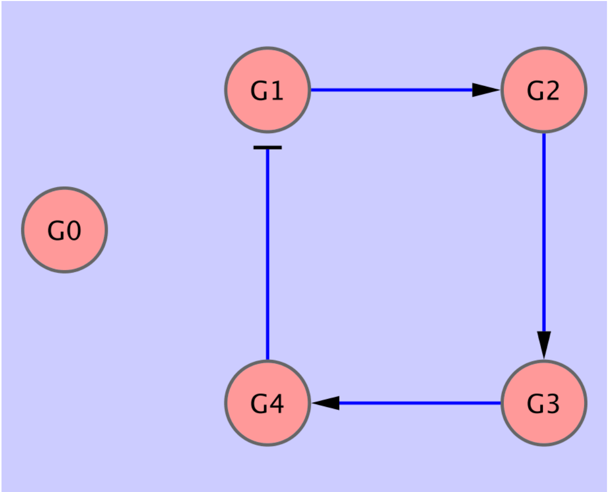

Figure 2: Very simple dynamic network consisting of 5 observables

If we run BNfinder on this data:

```
bnf -e input3.txt -n output3.sif -v
```

We can see that the program was unable to correctly reconstruct all the edges. Again, if we look at the statistics of edge occurences in suboptimal networks,

```
bnf -e input3.txt -n output3.sif -v -i 10 -t output3.txt
```

we can see that the correct edges are the most commonly occuring ones, but they score lower than empty parent sets.

In this case we can show how perturbational data can be integrated into this framework. We have collected gene expression from 5 time-series containing one single gene knockout for each of the genes: data/input4.txt. The perturbations are noted by including the following lines in the preamble of the data file:

```
#perturbed EXP1 G1
#perturbed EXP2 G2
#perturbed EXP3 G3
#perturbed EXP4 G4
#perturbed EXP5 G0
```

If we run BNfinder on the perturbed data, we can see that all the edges are reconstructed with high confidence.

```
bnf -e input4.txt -n output4.sif -v -i 10 -t output4.txt
```

Setting priors

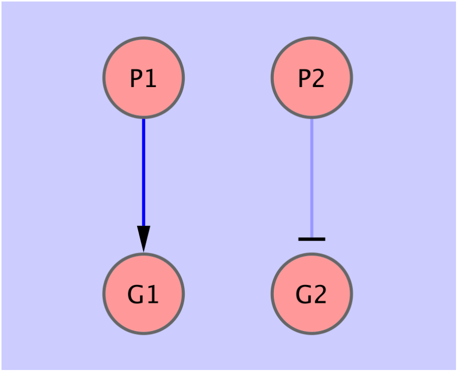

Figure 3: Exemplary network containing dependencies of different strength

In some cases it might be useful to include some prior information on the network structure into the process of inference. We will illustrate this on an example of a simple network similar to the one described in section 2.1. This time it is an even simpler network, with 2 conditions and 2 genes as depicted in Fig. 3. Even though topology of the network is very simple, the problem lies in the fact that the dependence of $G2$ on $P2$ is weaker than the dependence of $G1$ on $P1$. This is why if we run our software on the unmodified dataset data/input5.txt,

```
bnf -e input5.txt -n output5.sif -v  -i 10 -t output5.txt
```

we can see that the program is unable to recover the $P2 \to G2$ edge. However, if we expect that $G2$ is responding weakly to its regulators, we can increase the prior probability of $G2$ being regulated by any of the factors $P1, P2$ via decreasing its weight:

```
#prioredge G2 0.33 P2 P1
```

We can see the edge appearing in the result as expected (see data/input6a.txt):

```
bnf -e input6a.txt -n output6a.sif -v  -i 10 -t output6a.txt
```

Similarly, if we expect, that in general gene response to condition $P2$ is weaker, we may modify the prior probability of the condition $P2$ to be a regulator:

```
#priorvert 0.33 P2
```

The result of running BNFinder with this input (data/input6b.txt) is very similar to the previous one:

```
bnf -e input6b.txt -n output6b.sif -v  -i 10 -t output6b.txt
```

2.2 Examples of published datasets

In this section, we present two more realistic examples of published datasets used for inference of Bayesian networks. The first one consists of measurements of states of protein signalling network under different perturbations [11]. It's

been used to infer causal relationships in the form of static Bayesian network.

The second dataset comes from documentation of the Banjo package [12] which can be downloaded from (http://www.cs.duke.edu/∼amink/software/banjo). It consists of 2000 observations describing a relatively large dynamic network consisting of 20 nodes. It may be considered a benchmark of the efficiency of our algorithm.

The third dataset is converted from an example attached to the globalMIT software for Bayesian network reconstruction. It is similar to the second example as it is also generated from a dynamic Bayesian network and consists of 2000 observations of 20 variables. However, in this case the variables are much less interconnected and there are many self-regulatory loops.

PLEASE NOTE that these datasets are too large to be run through BNFinder webserver. If you would like to run them, please download the software.

Static Protein signalling network

In this section we present how BNfinder can be applied to a protein signalling network analyzed by Sachs et al [11]. We took the data from the article, and transformed it into the format suitable for BNfinder. We also needed to specify several properties of the data in the preamble of the file data/sachs.inp

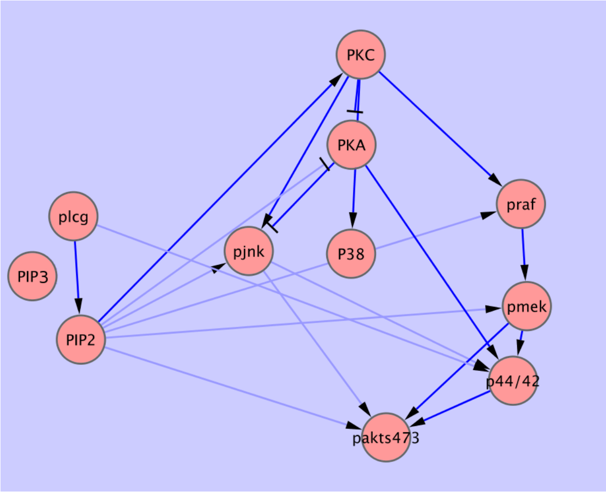

Figure 4: Reconstruction of the protein signalling network. Dark blue arrows represent dependencies found in literature. Light blue arrows represent dependencies found by BNfinder but not expected by Sachs et al. [11]

Firstly, we needed to specify that the data are continuous measurements:

```
#continuous praf pmek plcg PIP2 PIP3 p44/42 pakts473 PKA PKC P38 pjnk
```

Then, we needed to specify the expected layer structure of the signalling pathway we are studying:

```
#regulators plcg
#regulators PIP3
#regulators PIP2
#regulators PKC
#regulators PKA
#regulators praf
#regulators pjnk pmek P38
#regulators p44/42
#regulators pakts473
```

Then we needed to specify which of the proteins are affected by different perturbations.

```
#perturbed cd3cd28psitect_0 PIP2
#perturbed cd3cd28psitect_1 PIP2
#perturbed cd3cd28psitect_2 PIP2
...
#perturbed cd3cd28g0076_0 PKC
#perturbed cd3cd28g0076_1 PKC
#perturbed cd3cd28g0076_2 PKC
...
```

When we finally run the BNfinder:

```
bnf -e sachs.inp -n sachs.sif -v
```

We obtain the network presented in Fig. 4. As we can see, the topology is quite consistent with the literature data. Out of 17 expected edges, BNfinder recovers 11 correctly.

Dynamic Bayesian network

This is a dataset of substantial size which is used [14] to assess the performance of our inference algorithm. The input dataset (data/input7.txt) consists of 2000 measurements of 20 variables and it takes approximately 3 hours to compute it on a modern PC (2.4Ghz Intel Core 2 duo).

We can run BNfinder with the following command (note that we are using the `-l` option to limit the number of parents to 5):

```
bnf -e input7.txt -n output7.sif -v -l 5 -txt output7.txt
```

In Fig. 5 we can see part of the network reconstructed by BNfinder. All the edges reported by Banjo are also present in the optimal network (dark blue). The optimal network contains a number of additional edges, not reported by Banjo.

If you want to see how much faster the MDL algorithm is, you can also run BNfinder with the following command:

```
bnf -s MDL -e input7.txt -n output7mdl.sif -v -l 5 -txt output7mdl.txt
```

Dynamic network containing self-regulatory loops

In this example, we can utilize both the `-g 1` option for allowing self-regulations as well as the `-s MIT` option for using the MIT score.

```
bnf -s MIT -e input8.txt -n output8.sif -v -l 3 -txt output8.txt -c output8.cpd -g 1
```

One additional parameter that is unique to the MIT score is the significance level α of the χ^2 distribution (`-a`). The default level for α is .9999, but we can increase/decrease it if we want to see fewer/more edges respectively.

For example, setting the level alpha to a higher value should give us more edges in the result:

```
bnf -s MIT -e input8.txt -n output8.sif -v -l 3 -a .9  -g 1
```

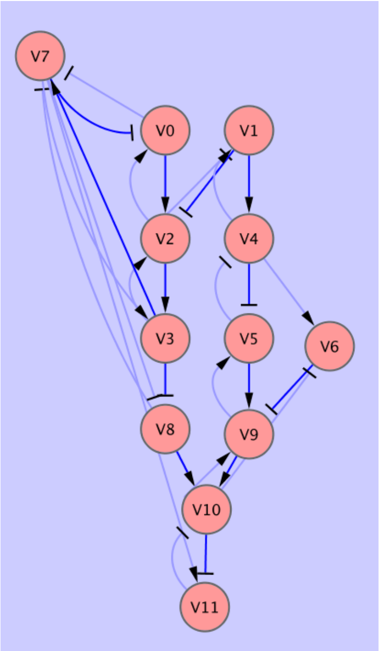

Figure 5: The optimal network reconstructed by BNfinder from the dynamic benchmark dataset. The edges reported also by Banjo are shown in dark blue.

Using multiple processors for faster computations

Since most current computers are equipped with multiple processors, we can take advantage of that fact to speed up BNFinder computation. Especially for large datasets, such as the ones described in previous sections, we can take full advantage of the parallell computation. For example, if we want BNfinder to run on 4 CPUs in parallell, we can use the `-k 4` option as in the following example:

```
bnf -s MIT -e input8.txt -n output8.sif -v -l 3 -a .9  -g 1 -k 4
```

2.3 Example of classification with BNfinder

`bnf-cv` and `bnc` tools can be used to solve classification tasks with classifier based on Bayesian networks. The former is used to perform a cross-validation test and the later to classify a dataset when you already have a classifier. In our example we will try to solve the following problem: we have points within the unit square; our positive set consists of those that are located in top-right and bottom-left corners, i.e. $x + y > 1.8$ or $x + y < 0.2$. The training set consists of 100 positive and 100 negative examples. They are visualised in the following Fig. 6. The data can be downloaded from here (data/training_set.txt). We marked x and y as continuous regulators. We will classify only one feature, but it is possible to perform cross-validation and classification procedure for more variables. All variables not marked as regulators are treated as variables to be explained by classifier.

To perform a 10-fold cross-validation we can use the following command:

```
bnf-cv -e training_set.txt -c net.cpd -k 10 -r ROC.pdf
```

As a result we obtain 10 files (net.cpd0, net.cpd1, ..., net.cpd9) containing networks in cpd format corresponding to respective folds of the cross-validation. Every execution of foregoing command will bring different results, because a split into 10 sets is done randomly. In the result file ROC.pdf there is a ROC plot showing the results of cross-validation

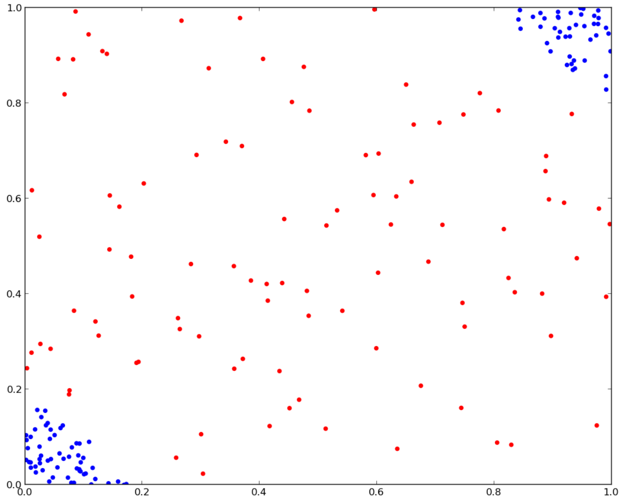

Figure 6: The training set used in the classification example. Positive examples are colored blue.

(see 7). Further results are printed to the standard output and contains (among others) information about regulators taken to each of 10 classifiers and AUC measure of each classifier's performance.

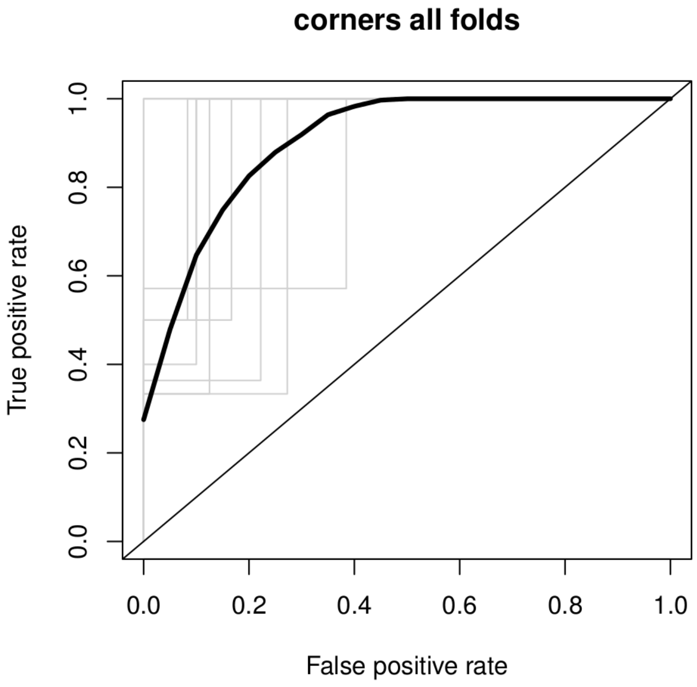

Figure 7: The Receiver operating characteristics curve for 10-fold cross-validation. The thick curve shows the average performance of classifiers.

To perform classification task on a test dataset we can use any of the nets obtained from cross-validation task but

usually it is better to train a classifier on the whole training dataset. It can be done by the following command:

```
bnf -e training_set.txt -c net.cpd
```

We will test out classifier on this (data/test_set.txt) dataset which consists of 1000 points from the unit square. Now, by using the `bnc` tool we can obtain the classification. In the Fig. 8 we can see the result of classifying our test dataset by classifier in the file net.cpd (we used 0.63 probability threshold to generate the plot):

```
bnc -o result.cls -p 1 -c net.cpd -d test_set.txt
```

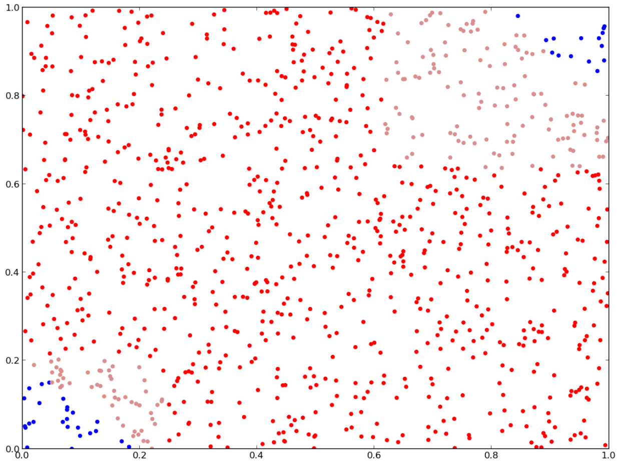

Figure 8: The result of classification. Blue and red points represent true positives and negatives. There was no false negatives. False positives are colored light red.

We can also find the most probable class for `corners` for every experiment by executing:

```
bnc -o result.cls -m 1 -c net.cpd -d test_set.txt
```

Finally, by executing

```
bnf-cv -e training_set.txt -k 1 -r ROC.pdf
```

command one can generate a colored plot of classifier trained on the whole training set. Different colors (explained on the right side of the picture) indicate probability thresholds above which we classify example as a positive one. An example of such a plot can be seen on the Fig. 9.

3 Supplementary methods

In the present section we give a brief exposition of the algorithm implemented in BNFinder and its computational cost for two generally used scoring criteria: Minimal Description Length and Bayesian-Dirichlet equivalence. For a fuller treatment, including detailed proofs, we refer the reader to [3, 4].

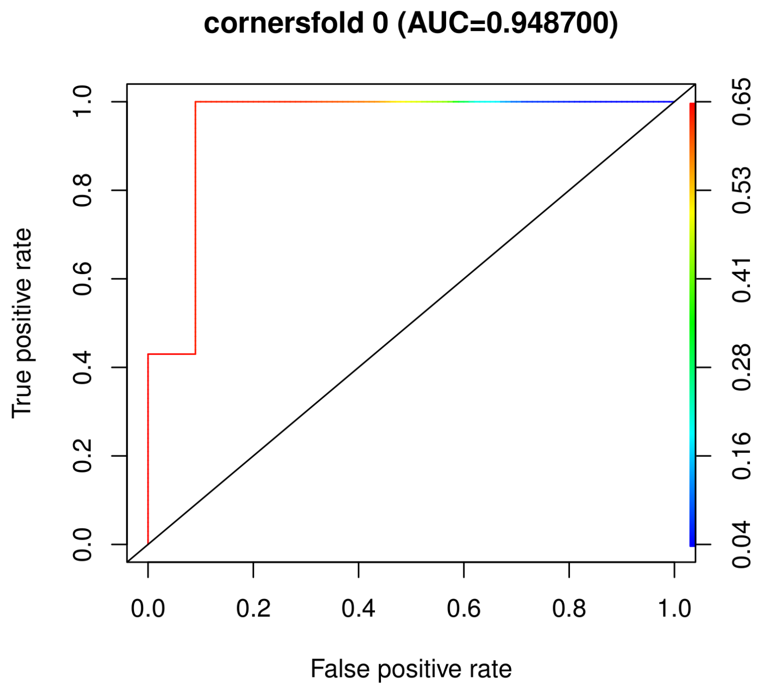

Figure 9: The effect of ploting ROC curve from 1-fold cross-validation.

3.1 Polynomial-time exact algorithm

A *Bayesian network* (BN) $\mathcal{N}$ is a representation of a joint distribution of a set of discrete random variables $\mathbf{X} = \{X_1, \ldots, X_n\}$. The representation consists of two components:

- a directed acyclic graph $\mathcal{G} = (\mathbf{X}, \mathbf{E})$ encoding conditional (in-)dependencies
- a family θ of conditional distributions $P(X_i|\mathbf{Pa}_i)$, where

$$\mathbf{Pa}_i = \{Y \in \mathbf{X} | (Y, X_i) \in \mathbf{E}\}$$

The joint distribution of $\mathbf{X}$ is given by

$$P(\mathbf{X}) = \prod_{i=1}^{n} P(X_i|\mathbf{Pa}_i) \tag{1}$$

The problem of learning a BN is understood as follows: given a multiset of $\mathbf{X}$-instances $\mathcal{D} = \{\mathbf{x}_1, \ldots, \mathbf{x}_N\}$ find a network graph $\mathcal{G}$ that best matches $\mathcal{D}$. The notion of a good match is formalized by means of a *scoring function* $S(\mathcal{G} : \mathcal{D})$ having positive values and minimized for the best matching network. Thus the point is to find a directed acyclic graph $\mathcal{G}$ with the set of vertices $\mathbf{X}$ minimizing $S(\mathcal{G} : \mathcal{D})$.

The BNFinder program is devoted to the case when there is no need to examine the acyclicity of the graph, for example:

- When dealing with *dynamic* Bayesian networks. A dynamic BN describes stochastic evolution of a set of random variables over discretized time. Therefore conditional distributions refer to random variables in neighboring time points. The acyclicity constraint is relaxed, because the ”unrolled” graph (with a copy of each variable in each time point) is always acyclic (see [6] for more details). The following considerations apply to dynamic BNs as well.

- In case of static Bayesian Networks, the user has to supply the algorithm with a partial ordering of the vertices, restricting the set of possible edges only to the ones consistent with the ordering. BNFinder lets the user to divide the set of variables into an ordered set of disjoint subsets of variables, where edges can only exist between variables from different subsets and they have to be consistent with the ordering. If such ordering is not known beforehand, one can try to run BNFinder with different orderings and choose a network with the best overall score.

In the sequel we consider some assumptions on the form of a scoring function. The first one states that $S(\mathcal{G}:\mathcal{D})$ decomposes into a sum over the set of random variables of *local scores*, depending on the values of a variable and its parents in the graph only.

Assumption 1 (additivity) $S(\mathcal{G}:\mathcal{D}) = \sum_{i=1}^{n} s(X_i, \mathbf{Pa}_i : \mathcal{D}|_{\{X_i\}\cup\mathbf{Pa}_i})$*, where* $\mathcal{D}|_{\mathbf{Y}}$ *denotes the restriction of* $\mathcal{D}$ *to the values of the members of* $\mathbf{Y} \subseteq \mathbf{X}$.

When there is no need to examine the acyclicity of the graph, this assumption allows to compute the parents set of each variable independently. Thus the point is to find $\mathbf{Pa}_i$ minimizing $s(X_i, \mathbf{Pa}_i : \mathcal{D}|_{\{X_i\}\cup\mathbf{Pa}_i})$ for each i.

Let us fix a dataset $\mathcal{D}$ and a random variable X. We denote by $\mathbf{X}'$ the set of potential parents of X (possibly smaller than $\mathbf{X}$ due to given constraints on the structure of the network). To simplify the notation we continue to write $s(\mathbf{Pa})$ for $s(X, \mathbf{Pa} : \mathcal{D}|_{\{X\}\cup\mathbf{Pa}})$.

The following assumption expresses the fact that scoring functions decompose into 2 components: g penalizing the complexity of a network and d evaluating the possibility of explaining data by a network.

Assumption 2 (splitting) $s(\mathbf{Pa}) = g(\mathbf{Pa}) + d(\mathbf{Pa})$ *for some functions* $g, d : \mathcal{P}(\mathbf{X}) \to \mathbb{R}^+$ *satisfying* $\mathbf{Pa} \subseteq \mathbf{Pa}' \implies g(\mathbf{Pa}) \leq g(\mathbf{Pa}')$.

This assumption is used in the following algorithm to avoid considering networks with inadequately large component g.

Algorithm 1

1. $\mathbf{Pa} := \emptyset$
2. *for each* $\mathbf{P} \subseteq \mathbf{X}'$ *chosen according to* $g(\mathbf{P})$
   (a) *if* $s(\mathbf{P}) < s(\mathbf{Pa})$ *then* $\mathbf{Pa} := \mathbf{P}$
   (b) *if* $g(\mathbf{P}) \geq s(\mathbf{Pa})$ *then return* $\mathbf{Pa}$*; stop*

In the above algorithm *choosing according to* $g(\mathbf{P})$ means choosing increasingly with respect to the value of the component g of the local score.

Theorem 1 *Suppose that the scoring function satisfies Assumptions 1-2. Then Algorithm 1 applied to each random variable finds an optimal network.*

A disadvantage of the above algorithm is that finding a proper subset $\mathbf{P} \subseteq \mathbf{X}'$ involves computing $g(\mathbf{P}')$ for all $\subseteq$-successors $\mathbf{P}'$ of previously chosen subsets. It may be avoided when a further assumption is imposed.

Assumption 3 (uniformity) $|\mathbf{Pa}| = |\mathbf{Pa}'| \implies g(\mathbf{Pa}) = g(\mathbf{Pa}')$.

The above assumption suggests the notation $\hat{g}(|\mathbf{Pa}|) = g(\mathbf{Pa})$. The following algorithm uses the uniformity of g to reduce the number of computations of the component g.

Algorithm 2

1. $\mathbf{Pa} := \emptyset$
2. *for* $p = 1$ *to* n
   (a) *if* $\hat{g}(p) \geq s(\mathbf{Pa})$ *then return* $\mathbf{Pa}$*; stop*
   (b) $\mathbf{P} = argmin_{\{\mathbf{Y} \subseteq \mathbf{X}' : |\mathbf{Y}| = p\}} s(\mathbf{Y})$
   (c) *if* $s(\mathbf{P}) < s(\mathbf{Pa})$ *then* $\mathbf{Pa} := \mathbf{P}$

Theorem 2 *Suppose that the scoring function satisfies Assumptions 1-3. Then Algorithm 2 applied to each random variable finds an optimal network.*

3.2 Minimal Description Length

The Minimal Description Length (MDL) scoring criterion originates from information theory [8]. A network $\mathcal{N}$ is viewed here as a model of compression of a dataset $\mathcal{D}$. The optimal model minimizes the total length of the description, i.e. the sum of the description length of the model and of the compressed data. MDL is effectively equivalent to Bayesian Information Criterion (BIC) (see [9]), which approximates Bayesian scores (see next section) and is also applicable to continuous data.

Let us fix a dataset $\mathcal{D} = \{\mathbf{x}_1, \ldots, \mathbf{x}_N\}$ and a random variable X. Recall the decomposition $s(\mathbf{Pa}) = g(\mathbf{Pa}) + d(\mathbf{Pa})$ of the local score for X. In the MDL score $g(\mathbf{Pa})$ stands for the length of the description of the local part of the network (i.e. the edges ingoing to X and the conditional distribution $P(X|\mathbf{Pa})$) and $d(\mathbf{Pa})$ is the length of the compressed version of X-values in $\mathcal{D}$.

Let k_Y denote the cardinality of the set $\mathcal{V}_Y$ of possible values of the random variable $Y \in \mathbf{X}$. Thus we have

$$g(\mathbf{Pa}) = |\mathbf{Pa}| \log n + \frac{\log N}{2}(k_X - 1) \prod_{Y \in \mathbf{Pa}} k_Y$$

where $\frac{\log N}{2}$ is the number of bits we use for each numeric parameter of the conditional distribution. This formula satisfies Assumption 2 but fails to satisfy Assumption 3. Therefore Algorithm 1 can be used to learn an optimal network, but Algorithm 2 cannot.

However, for many applications we may assume that all random variables have the same value set $\mathcal{V}$ of cardinality k. In this case we obtain the formula

$$g(\mathbf{Pa}) = |\mathbf{Pa}| \log n + \frac{\log N}{2}(k - 1)k^{|\mathbf{Pa}|}$$

which satisfies Assumption 3. For simplicity, we continue to work under this assumption.

Compression with respect to the network model is understood as follows: when encoding the X-values, the values of $\mathbf{Pa}$-instances are assumed to be known. Thus the optimal encoding length is given by

$$d(\mathbf{Pa}) = N \cdot H(X|\mathbf{Pa})$$

where $H(X|\mathbf{Pa}) = -\sum_{v \in \mathcal{V}} \sum_{\mathbf{v} \in \mathcal{V}^{\mathbf{Pa}}} P(v, \mathbf{v}) \log P(v|\mathbf{v})$ is the conditional entropy of X given $\mathbf{Pa}$ (the distributions are estimated from $\mathcal{D}$).

Since all the assumptions from the previous section are satisfied, Algorithm 2 may be applied to learn the optimal network. Let us turn to the analysis of its complexity.

Theorem 3 *The worst-case time complexity of Algorithm 2 for the MDL score is* $\mathcal{O}(n^{\log_k N} N \log_k N)$.

3.3 Bayesian-Dirichlet equivalence

The Bayesian-Dirichlet equivalence (BDe) scoring criterion originates from Bayesian statistics [1]. Given a dataset $\mathcal{D}$ the optimal network structure $\mathcal{G}$ maximizes the *posterior* conditional probability $P(\mathcal{G}|\mathcal{D})$. We have

$$P(\mathcal{G}|\mathcal{D}) \propto P(\mathcal{G})P(\mathcal{D}|\mathcal{G}) = P(\mathcal{G}) \int P(\mathcal{D}|\mathcal{G},\theta)P(\theta|\mathcal{G})d\theta$$

where $P(\mathcal{G})$ and $P(\theta|\mathcal{G})$ are *prior* probability distributions on graph structures and conditional distributions' parameters, respectively, and $P(\mathcal{D}|\mathcal{G},\theta)$ is evaluated due to (1).

Heckerman et al. [7], following Cooper and Herskovits [1], identified a set of independence assumptions making possible decomposition of the integral in the above formula into a product over $\mathbf{X}$. Under this condition, together with a similar one regarding decomposition of $P(\mathcal{G})$, the scoring criterion

$$S(\mathcal{G}:\mathcal{D}) = -\log P(\mathcal{G}) - \log P(\mathcal{D}|\mathcal{G})$$

obtained by taking $-\log$ of the above term satisfies Assumption 1. Moreover, the decomposition $s(\mathbf{Pa}) = g(\mathbf{Pa}) + d(\mathbf{Pa})$ of the local scores appears as well, with the components g and d derived from $-\log P(\mathcal{G})$ and $-\log P(\mathcal{D}|\mathcal{G})$, respectively.

The distribution $P((\mathbf{X},\mathbf{E})) \propto \prod_{e\in\mathbf{E}} \alpha_e$ with penalty parameters $0 < \alpha_e < 1$ is commonly used as a prior over the network structures. BNFinder sets $\alpha_{(Y,X)} = 1/k_Y$ by default. This choice results in the function

$$g(\mathbf{Pa}) = \sum_{Y\in\mathbf{Pa}} \log k_Y$$

satisfying Assumptions 2. If we moreover assume that all random variables have the same value set $\mathcal{V}$ of cardinality k, we obtain the function

$$g(\mathbf{Pa}) = |\mathbf{Pa}| \log k$$

satisfying also Assumption 3. For simplicity, we continue to work under this assumption.

However, it should be noticed that there are also used priors which satisfy neither Assumption 2 nor 3, e.g. $P(\mathcal{G}) \propto \alpha^{\Delta(\mathcal{G},\mathcal{G}_0)}$, where $\Delta(\mathcal{G},\mathcal{G}_0)$ is the cardinality of the symmetric difference between the sets of edges in $\mathcal{G}$ and in the prior network $\mathcal{G}_0$.

The *Dirichlet distribution* is generally used as a prior over the conditional distributions' parameters. It yields

$$d(\mathbf{Pa}) = \log\left(\prod_{\mathbf{v}\in\mathcal{V}^{|\mathbf{Pa}|}} \frac{\Gamma(\sum_{v\in\mathcal{V}}(H_{v,\mathbf{v}} + N_{v,\mathbf{v}}))}{\Gamma(\sum_{v\in\mathcal{V}} H_{v,\mathbf{v}})} \prod_{v\in\mathcal{V}} \frac{\Gamma(H_{v,\mathbf{v}})}{\Gamma(H_{v,\mathbf{v}} + N_{v,\mathbf{v}})}\right)$$

where Γ is the *Gamma* function, $N_{v,\mathbf{v}}$ denotes the number of samples in $\mathcal{D}$ with $X = v$ and $\mathbf{Pa} = \mathbf{v}$, and $H_{v,\mathbf{v}}$ is the corresponding *hyperparameter* of the Dirichlet distribution.

Setting all the hyperparameters to 1 yields

$$d(\mathbf{Pa}) = \log\left(\prod_{\mathbf{v}\in\mathcal{V}^{|\mathbf{Pa}|}} \frac{(k-1+\sum_{v\in\mathcal{V}} N_{v,\mathbf{v}})!}{(k-1)!} \prod_{v\in\mathcal{V}} \frac{1}{N_{v,\mathbf{v}}!}\right) =$$

$$= \sum_{\mathbf{v}\in\mathcal{V}^{|\mathbf{Pa}|}} \left(\log(k-1+\sum_{v\in\mathcal{V}} N_{v,\mathbf{v}})! - \log(k-1)! - \sum_{v\in\mathcal{V}} \log N_{v,\mathbf{v}}!\right)$$

where $k = |\mathcal{V}|$. For simplicity, we continue to work under this assumption (following Cooper and Herskovits [1]). The general case may be handled in a similar way.

The following result allows to refine the decomposition of the local score into the sum of the components g and d.

Proposition 1 *Define* $d_{min} = \sum_{v \in \mathcal{V}} (\log(k-1+N_v)! - \log(k-1)! - \log N_v!)$*, where* N_v *denotes the number of samples in* $\mathcal{D}$ *with* $X = v$*. Then* $d(\mathbf{Pa}) \geq d_{min}$ *for each* $\mathbf{Pa} \in \mathbf{X}$.

By the above proposition, the decomposition of the local score given by $s(\mathbf{Pa}) = g'(\mathbf{Pa}) + d'(\mathbf{Pa})$ with the components $g'(\mathbf{Pa}) = g(\mathbf{Pa}) + d_{min}$ and $d'(\mathbf{Pa}) = d(\mathbf{Pa}) - d_{min}$ satisfies all the assumptions required by Algorithm 2. Let us turn to the analysis of its complexity.

Theorem 4 *The worst-case time complexity of Algorithm 2 for the BDe score with the decomposition of the local score given by* $s(\mathbf{Pa}) = g'(\mathbf{Pa}) + d'(\mathbf{Pa})$ *is* $\mathcal{O}(n^{N \log_{\alpha^{-1}} k} N^2 \log_{\alpha^{-1}} k)$.

3.4 Mutual information test

The Mutual Information Test (MIT) scoring criterion originates from the concept of mutual information, belonging to the family of measures based on information theory [2]. Briefly speaking, this method combines mutual information measure and a statistical independence test based on the chi-square dustribution assosiated with it. The goodness of a fit of the particular network is computed as the total mutual information between each node and its parents. This score is then penalized by a term corresponding to the degree of statistical significance of the shared information.

Let $\mathcal{D}$ be a dataset with N observations, $\mathcal{G}$ be the dynamic bayesian network. Let $X = \{X_1, ..., X_n\}$ be the set of n variables, with each of it corresponding to $\{r_1, ..., r_n\}$ discrete states. Let's denote the set of parents of X_i in $\mathcal{G}$ with corresponding $\{r_{i1}, ..., r_{is_i}\}$ discrete states as $\mathbf{Pa}_i = \{X_{i1}, ..., X_{is_i}\}$. Then the MIT score is defined as follows [13]:

$$\mathcal{S}(\mathcal{G} : \mathcal{D}) = \sum_{i=0; \mathbf{Pa}_i \neq \emptyset}^{n} \{2N \cdot I(X_i, \mathbf{Pa}_i) - \sum_{j=1}^{s_i} \chi_{\alpha l_i \sigma_i(j)}\}$$

In this formula $I(X_i, \mathbf{Pa}_i)$ denotes the mutual information between X_i and its parents as estimated from $\mathcal{D}$ and defined as

$$I(X; Y) = \sum_{y \in Y} \sum_{x \in X} p(x, y) \log\left(\frac{p(x, y)}{p(x)p(y)}\right)$$

$\chi_{\alpha l_i \sigma_i(j)}$ is the chi-square distribution at significance level $1 - \alpha$. It is defined as the value such that

$$p(\chi^2(l_{ij}) \leq \chi_{\alpha l_i j}) = \alpha$$

The term $l_{i\sigma_i(j)}$ denotes the degrees of freedom and is defined as

$$l_{i\sigma_i(j)} = \begin{cases} (r_i - 1)(r_{i\sigma_i(j)} - 1) \prod_{k=1}^{j-1} r_{i\sigma_i(k)}, & j = 2.., s_i. \\ (r_i - 1)(r_{i\sigma_i(j)} - 1), & j = 1. \end{cases}$$

where $\sigma_i = \{\sigma_i(1), ..., \sigma_i(s_i)\}$ is any permutation of the index set $\{1...s_i\}$ of $\mathbf{Pa}_i$ such that, the number of states of the variables decreases with the increasing position in the permutation.

Recall the decomposition $S_{MIT}(\mathbf{Pa}_i) = d_{MIT}(\mathbf{Pa}_i) + g_{MIT}(\mathbf{Pa}_i)$. In this case:

$$d_{MIT}(\mathbf{Pa}_i) = 2N \cdot I(X_i, \mathbf{X}) - 2N \cdot I(X_i, \mathbf{Pa}_i)$$

$$g_{MIT}(\mathbf{Pa}_i) = \sum_{j=1}^{s_i} \chi_{\alpha l_i \sigma_i(j)}$$

Roughly, d_{MIT} measures the accuracy of representing the joint distribution of $\mathcal{D}$ by $\mathcal{G}$ while g_{MIT} measures the complexity of this representation. This decomposition satisfies Assumption 2. However, MIT score defined in this way does not satisfy Assumption 3. Therefore, we introduce an assumption that all the variables have the same number of discrete states.

Assumption 4 (uniformity) *All variables in X have the same number of discrete states k.*

Under this assumption it can be easily shown that g_{MIT} satisfies Assumption 3.

Theorem 5 *[10] The worst-case time complexity of Algorithm 2 for the MIT score under the assumption of the variables uniformity is polynomial in the number of variables.*

3.5 Continuous variables

All the scoring functions implemented in BNFinder (MDL, BDe and MIT) were originally designed for discrete variables. In order to avoid arbitrary discretization of continuous data we adapted them to deal with continuous variables directly. Moreover, our method works also with heterogenous data sets joining together discrete and continuous variables.

The distribution of each continuous variable X is assumed to be a mixture of two normal distributions. Mixture components correspond to the two possible values (*low* and *high*) of a related hidden discrete variable X' and X is viewed as its observable reflection. Consequently, the conditional distributions of X is given by:

$$P(X|\mathbf{Pa}) = \sum_{v \in \{low,high\}} \sum_{\mathbf{v} \in \{low,high\}^{|\mathbf{Pa}|}} P(X|X'=v)P(X'=v|\mathbf{Pa}'=\mathbf{v})P(\mathbf{Pa}'=\mathbf{v}|\mathbf{Pa})$$

Conditional distributions $P(X|X')$ are assumed to be independent for all variables X. Thus we estimate their parameters separately for each X in a preprocessing step. Estimation is based on data clustering with the *k-means* algorithm ($k = 2$, cutting the set of variable values in the median yields initial clusters). Due to the independence assumption, these parameters enable us to calculate also $P(\mathbf{Pa}'|\mathbf{Pa}) = \prod_{Y \in \mathbf{Pa}} P(Y'|Y)$. Thus the space of possible conditional distributions on continuous variables forms a family of Gaussian mixtures, parameterized by $P(X'|\mathbf{Pa}')$, conditional distributions on corresponding discrete variables.

From a technical point of view, BNFinder learns optimal network structures for these discrete variables, using scoring functions adapted to handle distributions on variable values instead of their determined values (expected values of original scores are computed). For continuous variables it gives optimal Bayesian networks from among all networks with conditional probability distributions belonging to the above defined family of Gaussian mixtures.

The following results present the complexity of our algorithm with continuous MDL and BDe scoring functions.

Theorem 6 *The worst-case time complexity of Algorithm 2 for the continuous MDL score is $\mathcal{O}(n^{\log N} N^2)$.*

Theorem 7 *The worst-case time complexity of Algorithm 2 for the continuous BDe score with the decomposition of the local score given by $s(\mathbf{Pa}) = g'(\mathbf{Pa}) + d'(\mathbf{Pa})$ is $\mathcal{O}((2n)^{\frac{N}{\log \alpha^{-1}}} N)$.*

3.6 Network density control

Recall that scoring functions decompose into 2 components: g penalizing the complexity of a network and d evaluating the possibility of explaining data by a network. The balance between these components influences the reliability of reconstructed relationships between variables – high g-to-d ratio results in high specificity, while low g-to-d ratio yields high sensitivity.

BNFinder has 3 mechanisms controlling this balance:

1. Option `-d` directly multiplies g-to-d ratio by a uniform factor for all pairs of variables.
2. Option `-r` sets g-to-d ratios for all edges according to specified proportion of false positive edges (type I error rate). It is particularly useful for heterogeneous sets of potential parents (continuous and discrete, discrete with varying levels of discretization), when different types of variables require specific treatment.

3. Input dataset preamble commands `#prioredge` and `#priorvert` modify g-to-d ratios for specified network edges. They are intended to incorporate into the learning process prior knowledge regarding possible variable dependencies. This method may be combined with one of previous mechanisms.

Option `-d` modifies g-to-d ratio by virtual dataset multiplication. Remaining two mechanisms adjust components g of the scoring function. It is done through redefining the formula for g by raising parameters k_Y, the number of discretization levels of a potential parent Y to appropriate powers $w_{Y,X}$ (in the case of BDe, it is just a modification of a prior distribution over network structures). Exponents $w_{Y,X}$ are either adjusted to required type I error rate or specified in the preamble of a dataset. They must satisfy $w_{Y,X} > 0$, default values $w_{Y,X} = 1$ result in the original formula for g component.

The control of type I error rate is based on a statistical model for 1-element set of potential parents and extrapolated to all sets. In the 1-element case there are only 2 potential parent sets: $\emptyset$ and $\{Y\}$, where Y is the only potential parent of considered regulated variable X. First, BNFinder calculates the required type I error probability for edge (Y, X). When no prior distribution on the network structure is specified in the dataset preamble, all edge error probabilities equal the requested type I error rate. Otherwise they are weighted according to the inverses of prior parameters.

Under a null hypothesis H_0 that variables X and Y are independent, type I error occurs when $s(\{Y\}) < s(\emptyset)$. We define $Z_{Y,X} = d(\{Y\}) - d(\emptyset)$ and $z_{Y,X} = g(\emptyset) - g(\{Y\})$. Thus $s(\{Y\}) < s(\emptyset)$ if and only if $Z_{Y,X} < z_{Y,X}$. Note that $Z_{Y,X}$ is a function of dataset values of random variables X and Y, so it is a random variable too. On the other hand, $z_{Y,X}$ is independent of the data and monotonically depends on $w_{Y,X}$.

BNFinder randomly permutes values of Y in the dataset and calculates $Z_{Y,X}$ for each permutation. The number of permutations is chosen according to requested type I error probability and the dataset size. Moreover, it may be manually shrunk to avoid exhaustive computations. The estimate of cumulative distribution function for $Z_{Y,X}$ under H_0 assumption is derived from calculated values and $d_{min} - d(\emptyset)$, the lower bound on $Z_{Y,X}$. Based on this distribution BNFinder adjusts $w_{Y,X}$ to yield $P(Z_{Y,X} < z_{Y,X}|H_0)$ equal to the required type I error probability for edge (Y, X).

References

[1] Gregory F. Cooper and Edward Herskovits. A Bayesian method for the induction of probabilistic networks from data. *Machine Learning*, 9:309–347, 1992.

[2] Luis M. de Campos. A scoring function for learning bayesian networks based on mutual information and conditional independence tests. *Journal of Machine Learning Research*, 7:2149–2187, 2006.

[3] Norbert Dojer. Learning Bayesian Networks Does Not Have to Be NP-Hard. In Rastislav Královic and PawełUrzyczyn, editors, *Proceedings of Mathematical Foundations of Computer Science 2006*, pages 305–314. Springer-Verlag, 2006. LNCS 4162.

[4] Norbert Dojer. An efficient algorithm for learning bayesian networks from data. *Fundamenta Informaticae*, 103(1):53–67, January 2010.

[5] Norbert Dojer, Anna Gambin, Bartek Wilczyński, and Jerzy Tiuryn. Applying dynamic Bayesian networks to perturbed gene expression data. *BMC Bioinformatics*, 7:249, 2006.

[6] N Friedman, K Murphy, and S Russell. Learning the structure of dynamic probabilistic networks. In G F Cooper and S Moral, editors, *Proceedings of the Fourteenth Conference on Uncertainty in Artificial Inteligence*, pages 139–147, 1998.

[7] David Heckerman, Dan Geiger, and David Maxwell Chickering. Learning Bayesian networks: The combination of knowledge and statistical data. *Machine Learning*, 20(3):197–243, Sep. 1995.

[8] W. Lam and F. Bacchus. Learning Bayesian belief networks: An approach based on the MDL principle. *Computational Intelligence*, 10(3):269–293, 1994.

[9] Richard E. Neapolitan. *Learning Bayesian Networks*. Prentice Hall, 2003.

[10] Vinh Nguyen. The globalmit toolkit for learning optimal dynamic bayesian network user guide. 2011.

[11] K. Sachs, O. Perez, D. Pe'er, D.A. Lauffenburger, and G.P. Nolan. Causal protein-signaling networks derived from multiparameter single-cell data. *Science*, 308:523–529, Apr 2005.

[12] V. Anne Smith, Jing Yu, Tom V Smulders, Alexander J Hartemink, and Erich D Jarvis. Computational inference of neural information flow networks. *PLoS Comput Biol*, 2(11):e161, Nov 2006.

[13] Nguyen Xuan Vinh, Madhu Chetty, Ross Coppel, and Pramod P.Wangikar. Globalmit: learning globally optimal dynamic bayesian network with the mutual information test criterion. *Bioinformatics*, 27(19):2765–2766, 2011.

[14] B. Wilczyński and N. Dojer. Bnfinder: exact and efficient method for learning bayesian networks. *Bioinformatics*, 25(2):286, 2009.
