# Supplementary figures and images for: Distributed Bayesian networks reconstruction on the whole genome scale
Source: PeerJ. 2018 Oct 19;6:e5692. doi: 10.7717/peerj.5692 (PMC6197044; doi:10.7717/peerj.5692)

**cornersfold 0 (AUC=0.948700)**

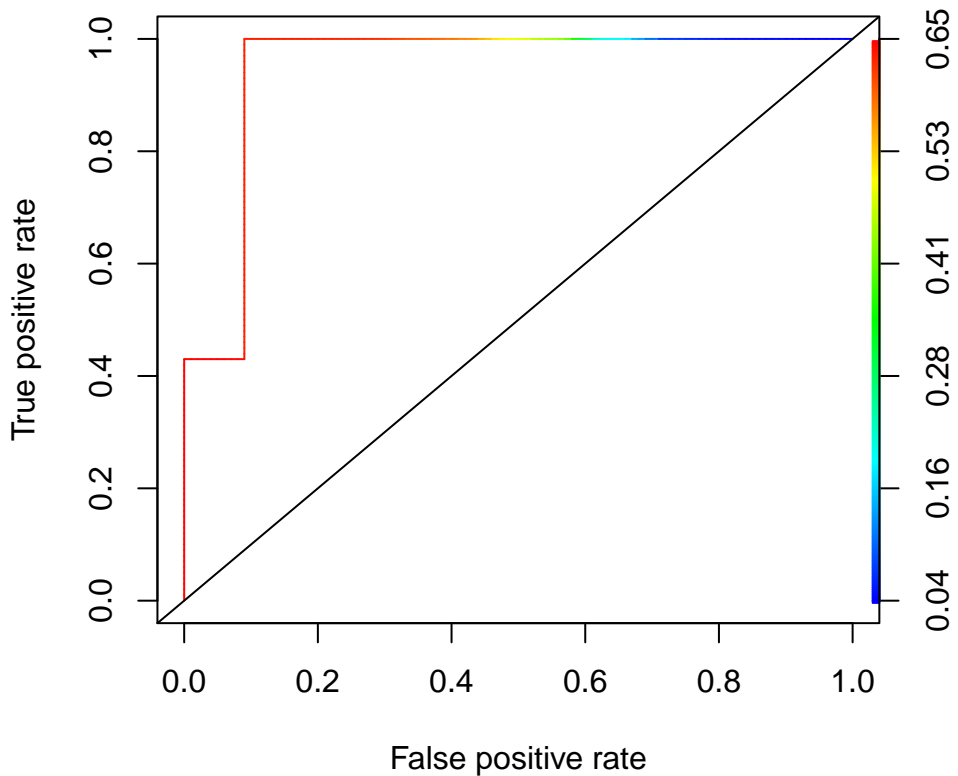

Supplement: Supplemental Information 1 [file peerj-06-5692-s001.zip › bnfinder-2.2/doc/img/ROC-K1.pdf]

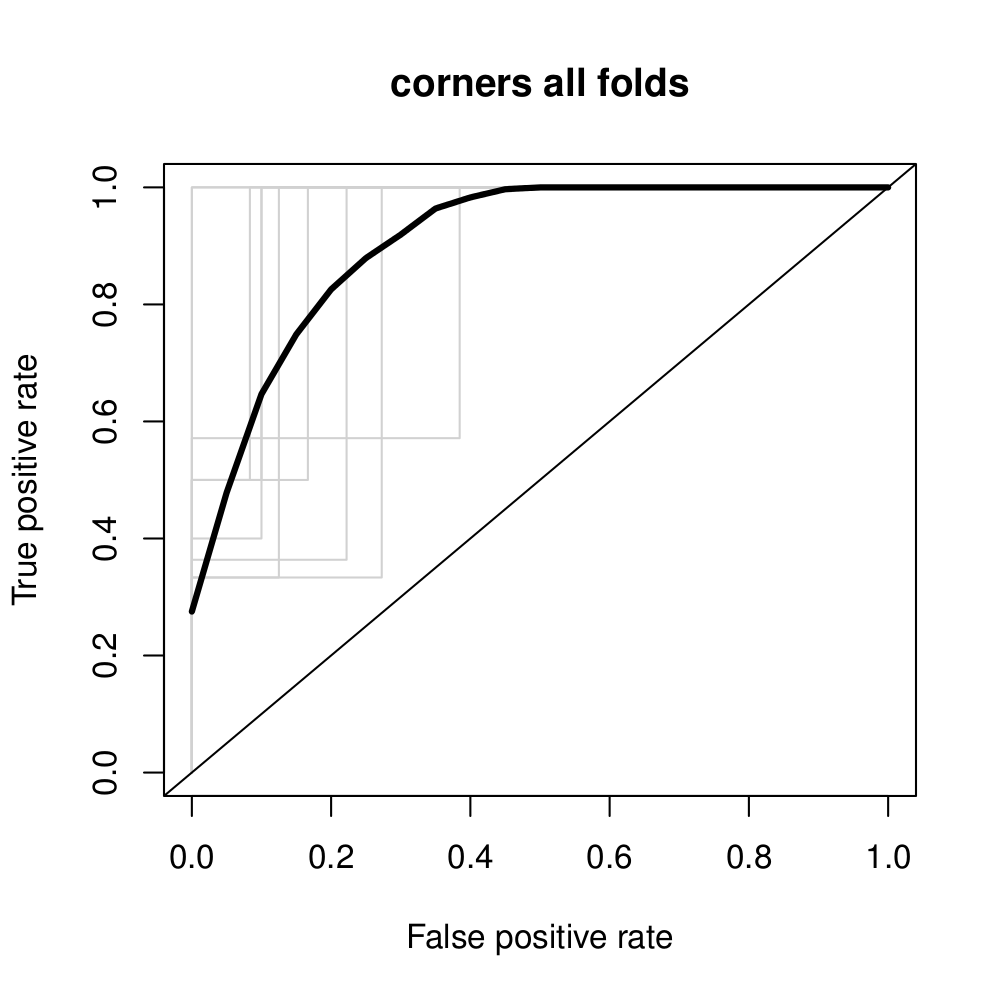

Supplement: Supplemental Information 1 [file peerj-06-5692-s001.zip › bnfinder-2.2/doc/img/ROC.png]

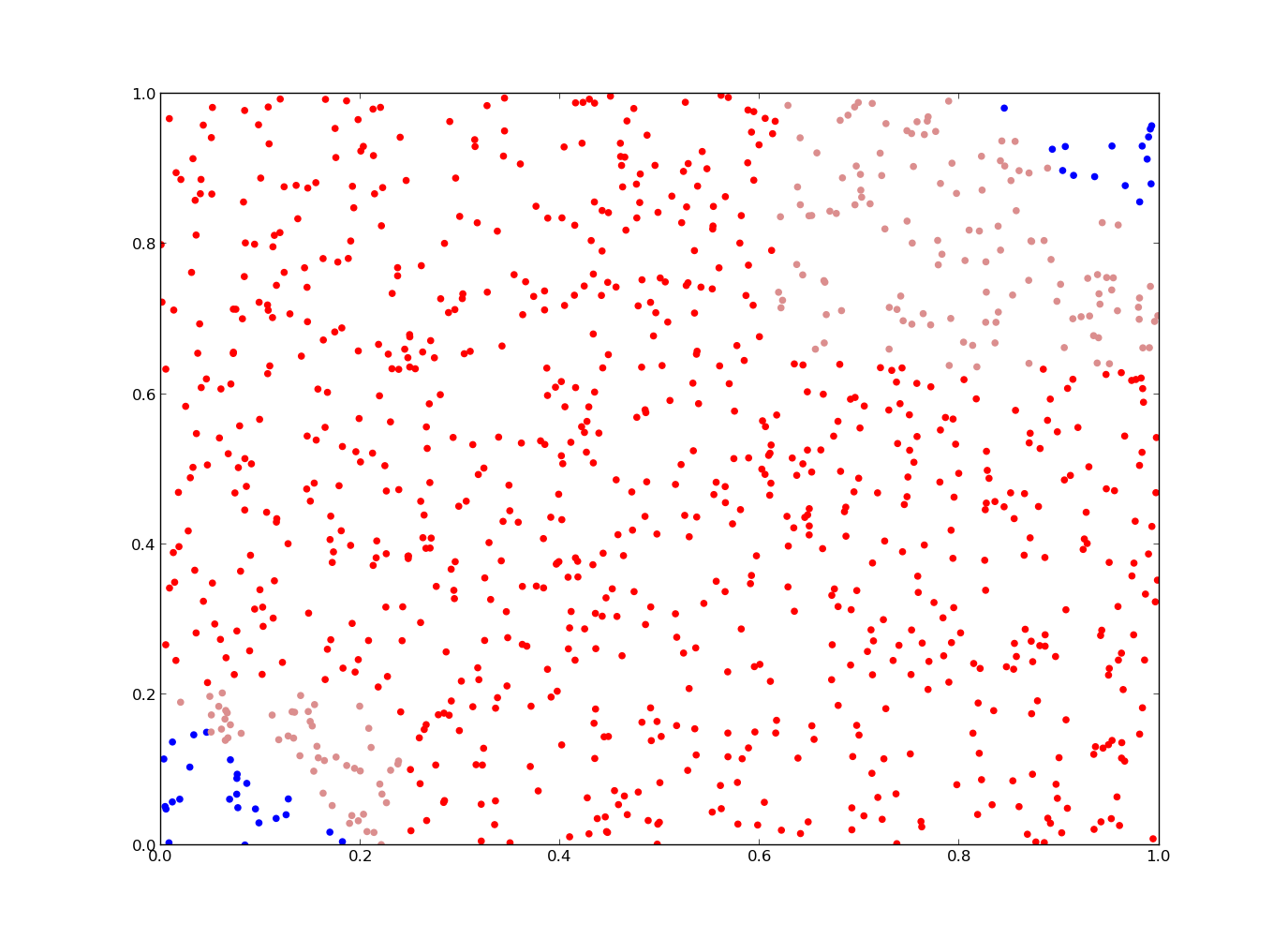

Supplement: Supplemental Information 1 [file peerj-06-5692-s001.zip › bnfinder-2.2/doc/img/classificationresult.png]

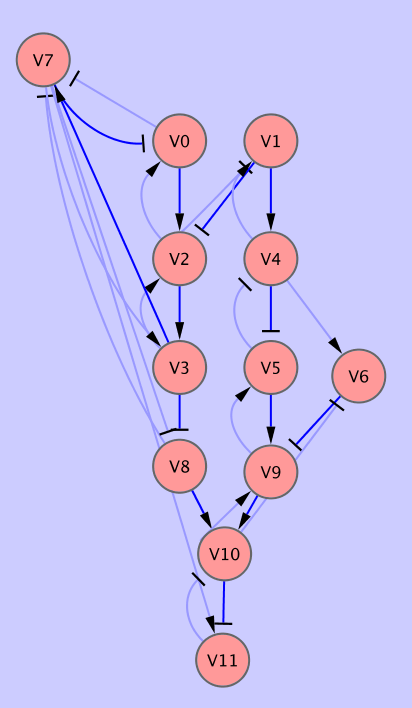

Supplement: Supplemental Information 1 [file peerj-06-5692-s001.zip › bnfinder-2.2/doc/img/dynamic.png]

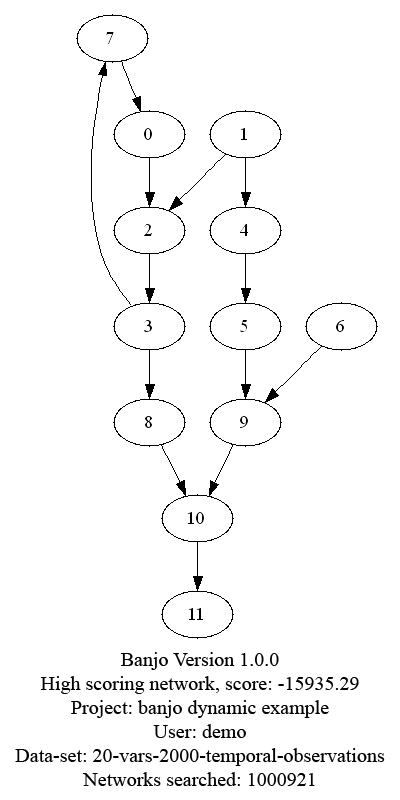

Supplement: Supplemental Information 1 [file peerj-06-5692-s001.zip › bnfinder-2.2/doc/img/dynamic2.png]

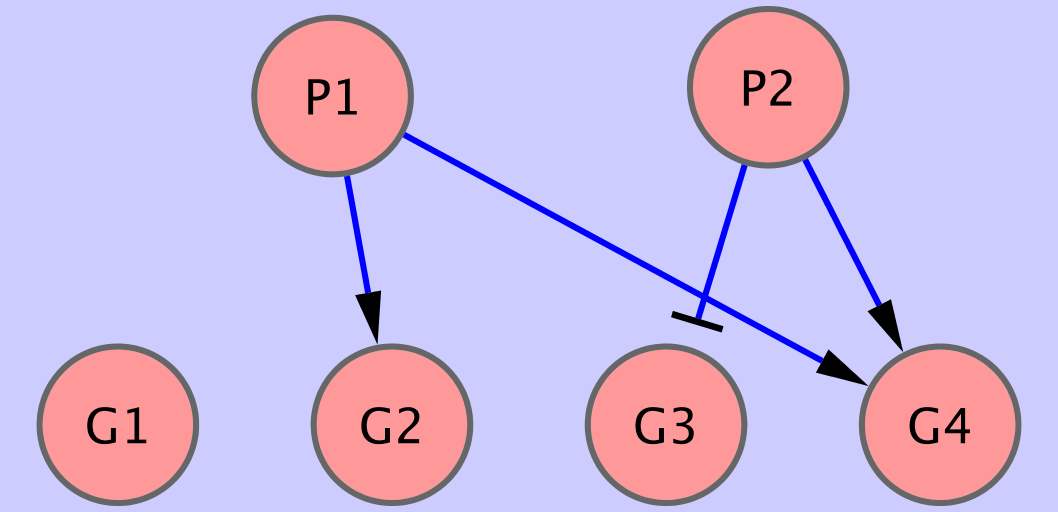

Supplement: Supplemental Information 1 [file peerj-06-5692-s001.zip › bnfinder-2.2/doc/img/network1.png]

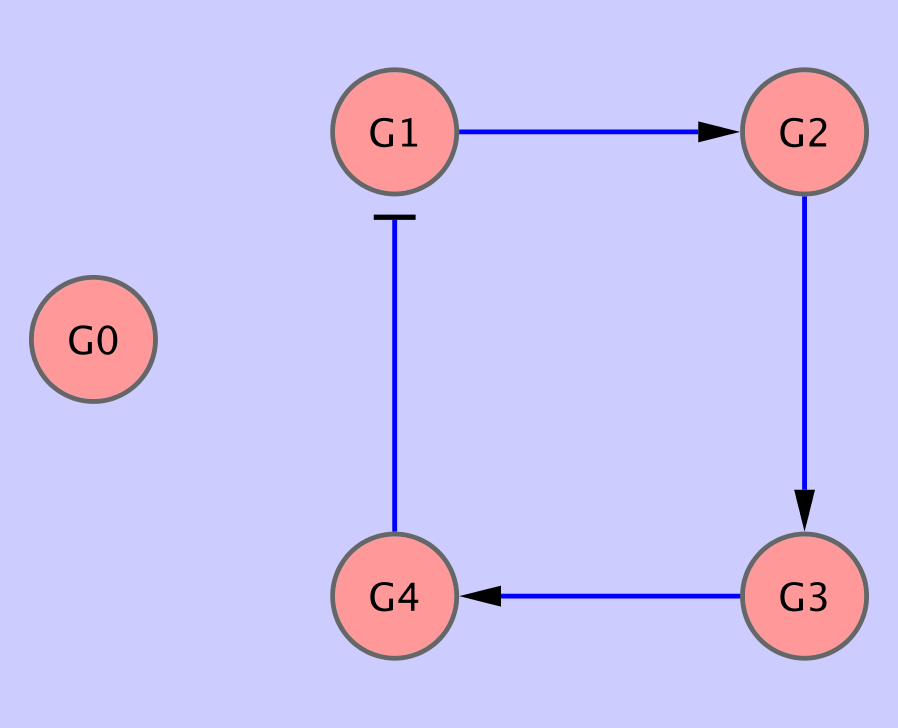

Supplement: Supplemental Information 1 [file peerj-06-5692-s001.zip › bnfinder-2.2/doc/img/network2.png]

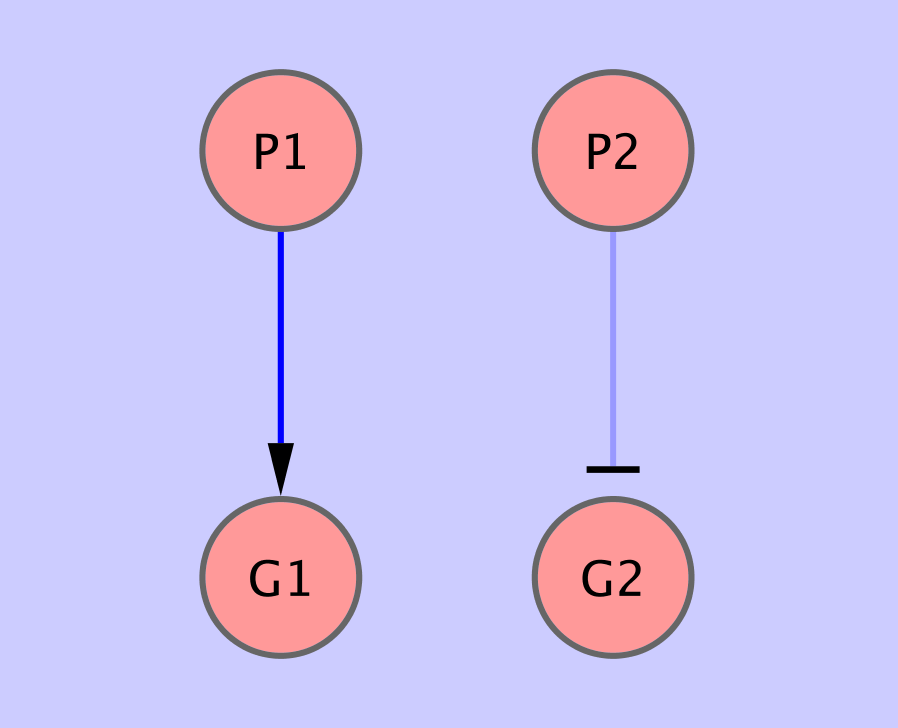

Supplement: Supplemental Information 1 [file peerj-06-5692-s001.zip › bnfinder-2.2/doc/img/network3.png]

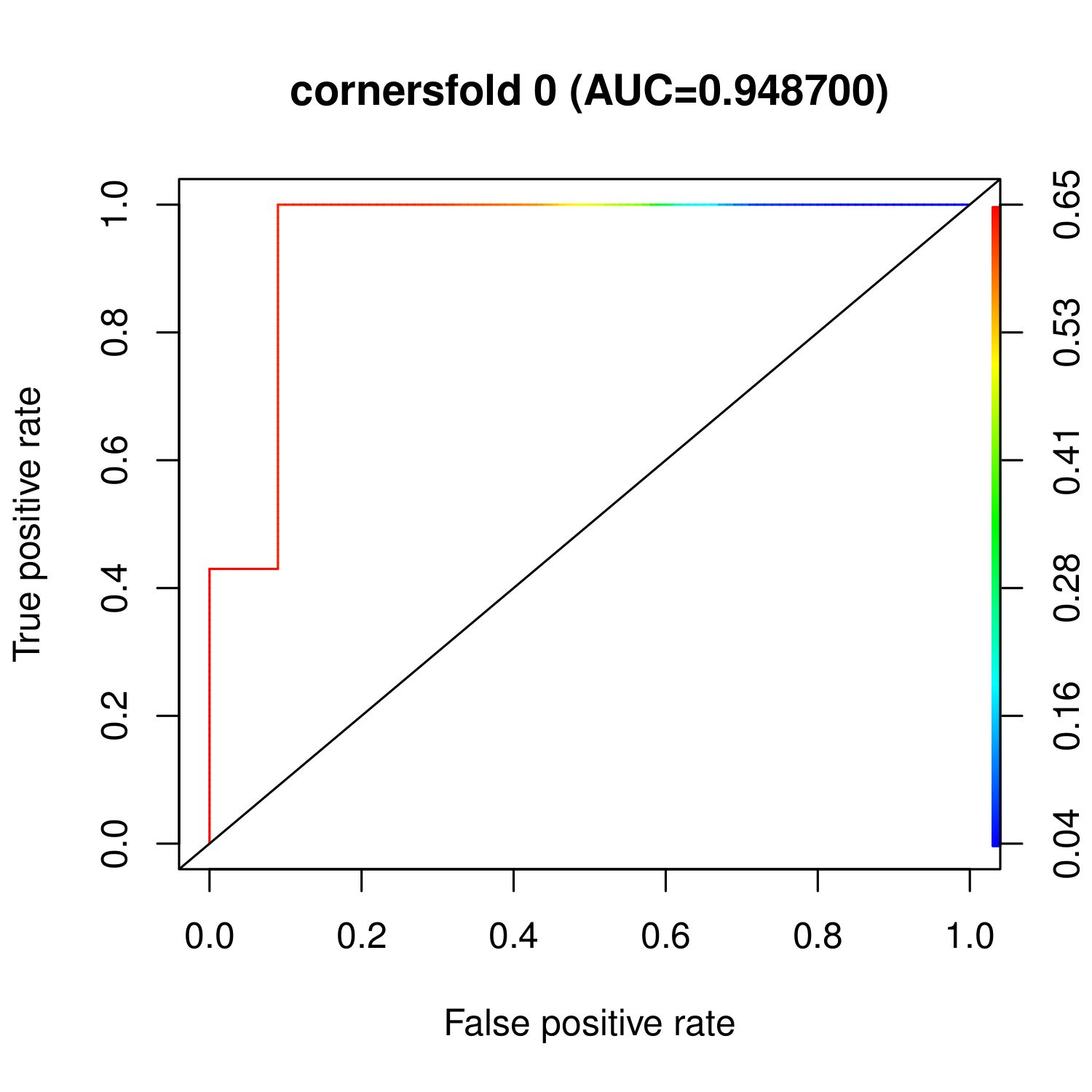

Supplement: Supplemental Information 1 [file peerj-06-5692-s001.zip › bnfinder-2.2/doc/img/rock1.png]

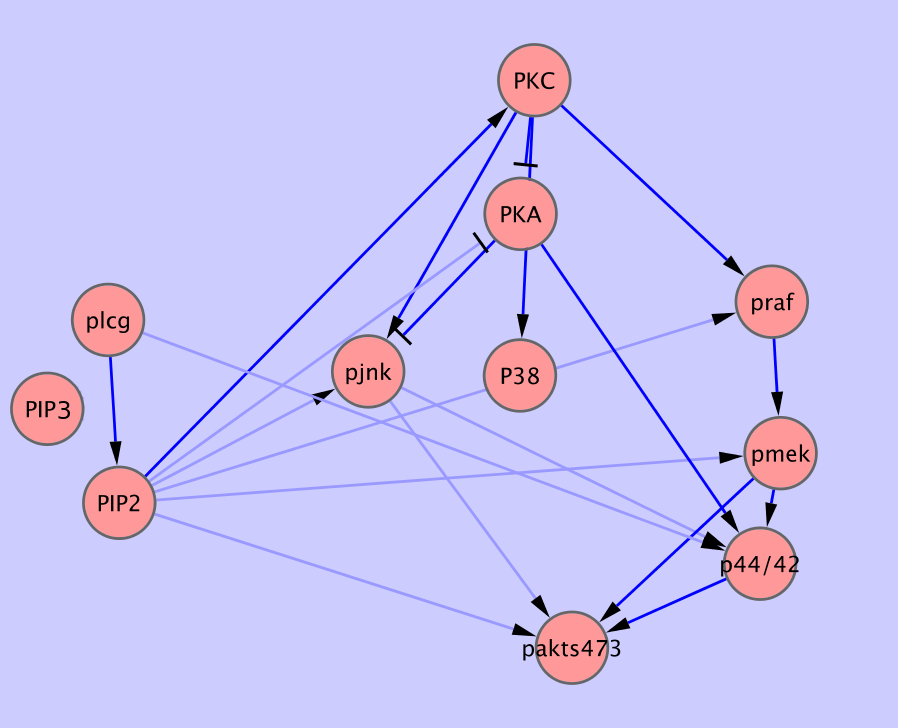

Supplement: Supplemental Information 1 [file peerj-06-5692-s001.zip › bnfinder-2.2/doc/img/static.png]

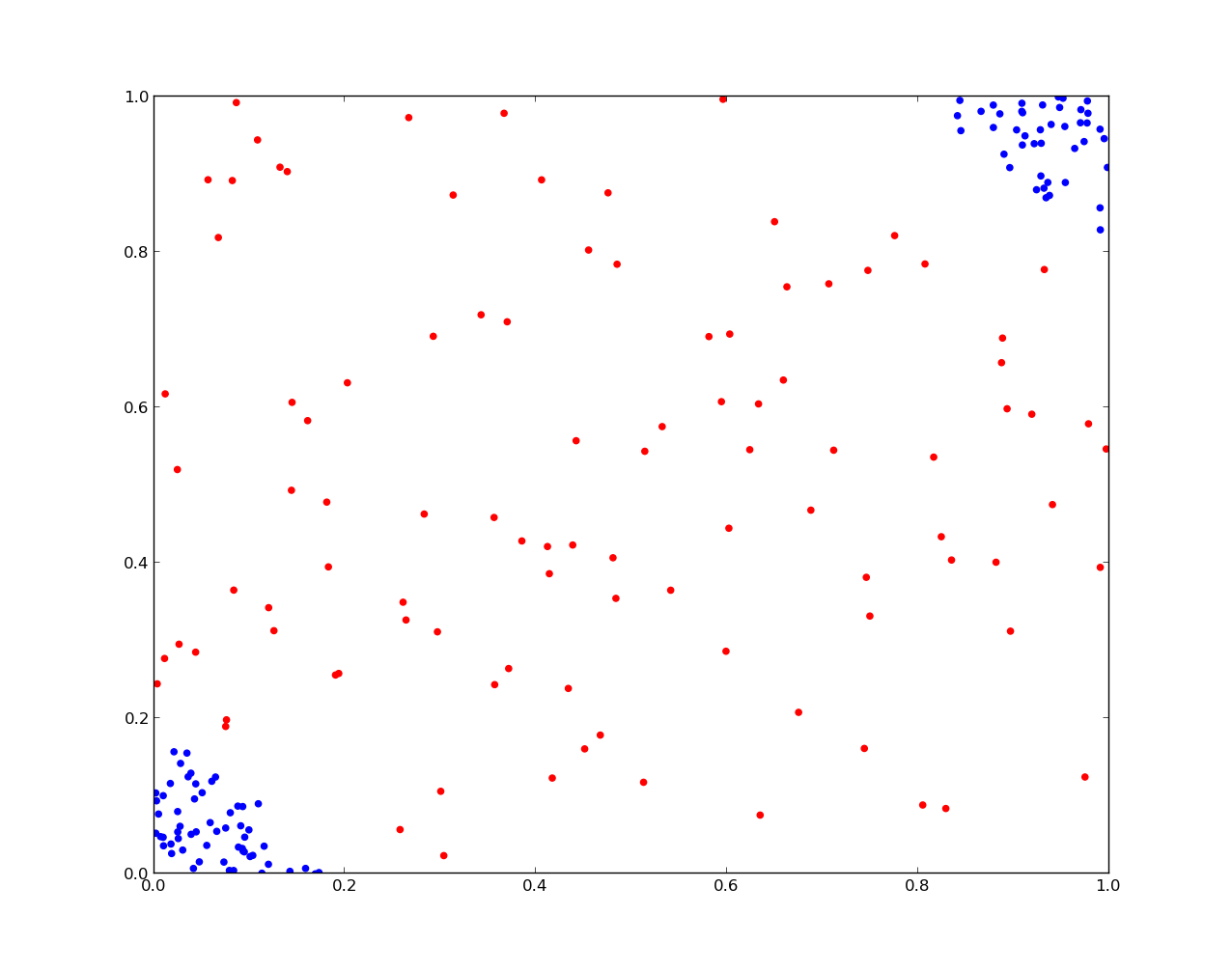

Supplement: Supplemental Information 1 [file peerj-06-5692-s001.zip › bnfinder-2.2/doc/img/training.png]
